# Defect Physics and Nanoscale Passivation Strategies in BaSi_2_ Thin-Film Photovoltaics

**DOI:** 10.3390/nano15231750

**Published:** 2025-11-21

**Authors:** Xiqiu Wang, Yehua Tang, Kaitao Xin, Liping Pan, Weiping Lu

**Affiliations:** School of Electrical and Energy Engineering, Nantong Institute of Technology, Nantong 226002, China; 20246143@ntit.edu.cn (X.W.); ktxinmiao@163.com (K.X.);

**Keywords:** BaSi_2_, thin films, defect physics, passivation, photovoltaic materials

## Abstract

Barium disilicide (BaSi_2_) was identified as a promising silicon-based photovoltaic absorber due to its near-optimal bandgap, strong optical absorption, and earth-abundant composition. However, the performance of BaSi_2_ thin-film solar cells was severely restricted by structural defects and interfacial instabilities that introduced localized electronic states and facilitated non-radiative recombination. These imperfections degraded carrier lifetime, mobility, and open-circuit voltage. This review systematically examined the formation, energetics, and electronic roles of intrinsic and extrinsic defects in BaSi_2_ thin films, and evaluated nanoscale passivation strategies developed to mitigate defect-induced losses. Chemical, dielectric, and interfacial approaches were critically analyzed with emphasis on their underlying mechanisms, limitations, and integration potential. The convergence of in situ characterization, first-principles modeling, and data-driven process optimization was expected to enable predictive defect control and rational interface design, thereby advancing BaSi_2_-based photovoltaics toward practical implementation.

## 1. Introduction

Silicon-based photovoltaics have long dominated the global solar market because of their mature fabrication technologies and high operational stability [1,2]. However, the indirect bandgap of crystalline silicon restricts optical absorption and limits the achievable power conversion efficiency [3]. To overcome these limitations, new material systems have been widely explored. One-dimensional (1D) nanostructures, such as nanowires and nanotubes, enhance light trapping and charge separation, making them promising for advanced solar cells [4]. Two-dimensional (2D) materials also offer tunable band structures and strong excitonic effects. Their electronic properties can be adjusted through composition and lattice engineering, leading to improved photovoltaic response [5]. These developments underscore the need for silicon-compatible materials that combine strong optical absorption, adjustable electronic structures, and compatibility with existing fabrication processes, such as barium disilicide (BaSi_2_) [6].

**Figure 1:**
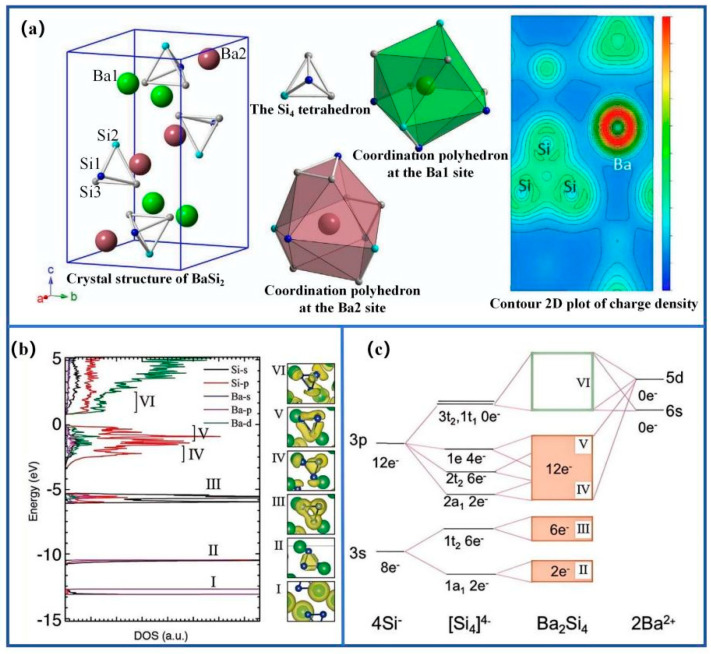
Structural and electronic characteristics of BaSi2: (**a**) orthorhombic crystal lattice with alternating Ba and Si double layers; (**b**) total and partial density of states with corresponding charge distribution; (**c**) molecular orbital configuration illustrating Ba-Si hybridization. Reproduced with permission. Refs. [7,8] Copyright 2020, *JJAP* & Copyright 2017, *J. Mater. Chem. A*.

**Figure 2:**
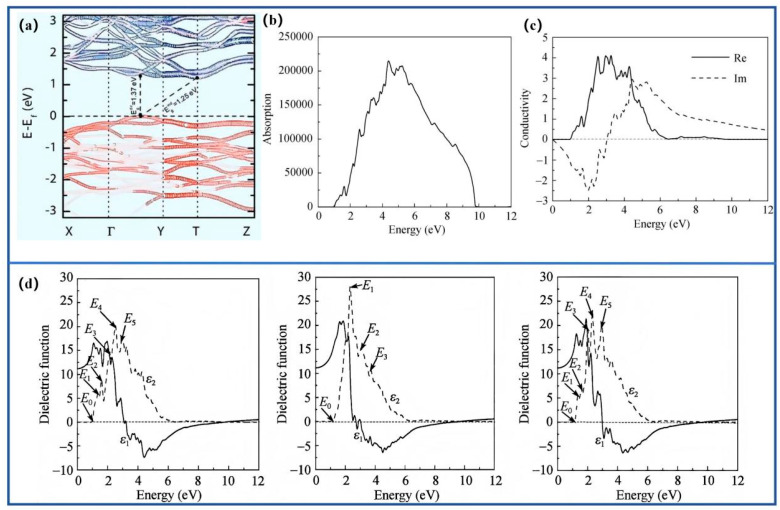
Electronic and optical anisotropy of BaSi2: (**a**) calculated band structure; (**b**) absorption coefficient showing direct bandgap transition; (**c**) electrical conductivity as a function of photon energy; (**d**) direction-dependent dielectric function along crystallographic axes. Reproduced with permission. Refs. [8,9] Copyright 2017, J. Mater. Chem. A, & Copyright 2009, Sci China Ser G-Phys Mech Astron.

Barium disilicide (BaSi_2_) possesses a direct bandgap of approximately 1.3 eV, a high absorption coefficient exceeding 10^5^ cm^−1^, and is composed of earth-abundant elements. Its orthorhombic lattice consists of alternating Ba and Si double layers, forming a covalent-ionic hybrid network that induces anisotropic electronic transport and strong light absorption (Figure 1a) [7]. Density-of-states analysis indicates that the valence band mainly originates from Si 3p orbitals, whereas the conduction band minimum arises from Ba 5d-Si 3p hybridization (Figure 1b,c) [8]. As shown in Figure 2, BaSi_2_ exhibits a direct bandgap near 1.3 eV with orientation-dependent dielectric response and anisotropic carrier mobility [8,9,10,11].

Despite their favorable intrinsic properties, BaSi_2_ thin films often contained intrinsic and epitaxial defects, including vacancies, interstitials, antisites, grain boundaries, and impurity complexes [12,13,14,15]. Their type and distribution were strongly governed by synthesis conditions. Molecular beam epitaxy (MBE) enabled precise control of stoichiometry and crystal orientation but was sensitive to Ba/Si flux variations, which could generate Ba-rich or Si-deficient regions. Magnetron sputtering was scalable yet tended to produce polycrystalline films with higher defect densities, which could be partially reduced by post-annealing at 600–750 °C. Chemical vapor deposition (CVD) and its variants, such as PECVD and MOCVD, offered good surface conformity and silicon compatibility, though impurity incorporation had to be carefully managed. Thus, the growth technique and processing parameters critically determined the intrinsic defect landscape, providing the physical basis for subsequent defect formation and electronic behavior.

These defects introduce deep electronic states that act as non-radiative recombination centers, reducing carrier lifetime, diffusion length, and open-circuit voltage. First-principles calculations and advanced characterization techniques have provided insights into defect energetics and recombination dynamics. However, a complete understanding of defect formation, migration, and interaction within the anisotropic lattice remains incomplete [16,17,18].

This review focuses on the defect physics and passivation strategies of BaSi_2_ thin films. It integrates first-principles calculations with experimental analyses to link defect behavior across multiple length scales to photovoltaic performance. Chemical, dielectric, and interfacial passivation approaches are critically evaluated in terms of their mechanisms, effectiveness, and integration challenges. The review concludes with perspectives on current limitations and future research opportunities for achieving defect-tolerant BaSi_2_-based photovoltaics.

Outline of the Review: To facilitate the readability and logical flow of this review, a brief overview of its structure is provided below. Section 2 discusses the formation, classification, and electronic effects of intrinsic and extrinsic defects in BaSi_2_ thin films, emphasizing their roles in carrier recombination and transport. Section 3 summarizes nanoscale passivation and defect-control strategies, including chemical, dielectric, and interfacial approaches, and evaluates their mechanisms, effectiveness, and practical challenges. Section 4 concludes with a perspective on current limitations, future research directions, and opportunities for integrating predictive modeling with in situ characterization to achieve defect-tolerant BaSi_2_ photovoltaics.

## 2. Defect Physics in BaSi_2_ Thin Films

### 2.1. Formation and Classification of Defects

**Figure 3:**
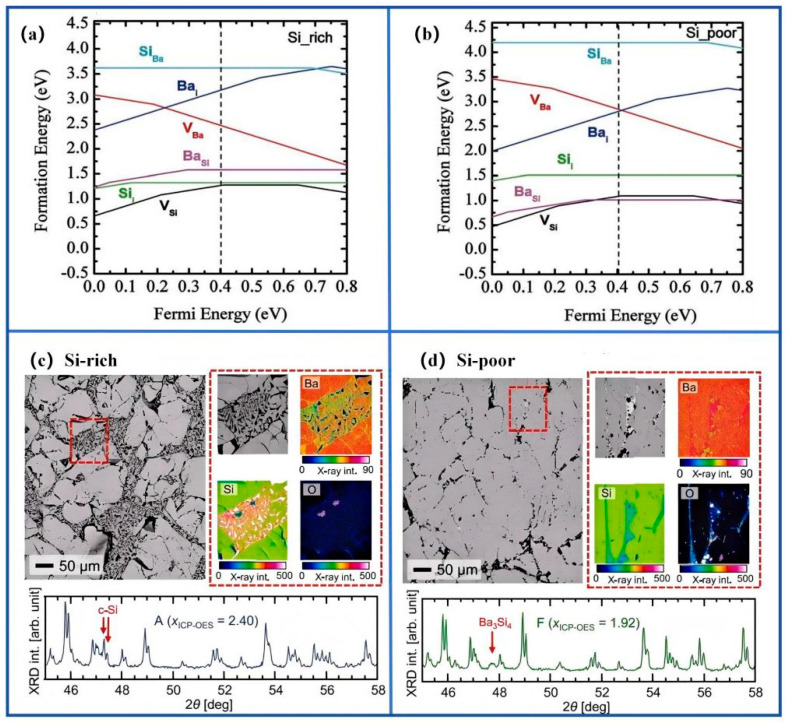
Correlation between intrinsic defect energetics and microstructural characteristics of BaSi_2_: (**a**,**b**) calculated formation energies of intrinsic point defects under Si-rich and Si-poor conditions; (**c**,**d**) experimental validation through XRD, BSE, and elemental mapping of Si-rich and Ba-rich samples. Reproduced with permission. Refs. [7,29] Copyright 2020, JJAP & Copyright 2024, ACTA MATER.

The performance of BaSi_2_ thin films was primarily determined by the type, density, and electronic activity of crystallographic defects. These defects originated from deposition thermodynamics, growth kinetics, and post-deposition treatments [19,20,21,22]. They were generally classified into point, extended, and interface defects, each exerting distinct effects on carrier dynamics. Point defects included vacancies, interstitials, and antisites, whose formation energies were strongly dependent on the local chemical potential. Recent first-principles studies by Mukesh Kumar et al. [7,8] systematically examined the intrinsic defect energetics of BaSi_2_ (Figure 3a,b). Hybrid DFT calculations revealed that under Si-rich conditions, silicon vacancies (V_Si_) were thermodynamically favored, introducing deep levels about 0.3–0.4 eV below the conduction band minimum, whereas Ba vacancies (V_Ba_) predominated under Ba-rich conditions. These results were consistent with the experimental observations of Kido and Koitabashi [6,12], who reported that Ba-rich sputtering caused lower photoresponsivity due to deep-level defects related to Ba interstitials and antisites. This agreement between computational and experimental results highlighted the critical influence of growth stoichiometry on the defect landscape of BaSi_2_ thin films. These defects acted as effective Shockley-Read-Hall (SRH) recombination centers, reducing minority-carrier lifetime. Barium vacancies (V_Ba_) and BaSi antisites produced shallow acceptor states that modulated carrier concentration and mobility [23,24,25].

Extended defects, including dislocations, stacking faults, and grain boundaries, disrupted lattice periodicity and generated localized strain and dangling bonds that acted as continuous pathways for non-radiative recombination. Some grain boundaries were electrically benign, but their interaction with point defects could significantly enhance recombination losses [26,27,28]. Correlating first-principles defect energetics with microstructural observations (XRD, BSE/EDS, TEM) established the relationship between synthesis conditions, defect formation, and electronic activity (Figure 3c,d) [29]. The calculated formation energies under different chemical potentials corresponded well with experimentally observed defect populations. Si-rich conditions favored V_Si_ formation. BSE/EDS mapping confirmed the presence of Si vacancies and local compositional inhomogeneity. These findings demonstrated that deposition conditions controlled defect types, densities, and associated electronic activity. A multi-modal analytical approach, combining PL spectroscopy, DLTS, and KPFM, provided a comprehensive framework for evaluating defect behavior. Although theoretical predictions and experimental observations were generally consistent, most models relied on equilibrium assumptions and lacked kinetic validation. The scarcity of in situ data further limited the accuracy of defect-growth correlations. Future studies should integrate dynamic simulations with real-time monitoring to establish a more predictive framework for defect control.

### 2.2. Electronic Structure Modulation and Carrier Dynamics

Defect-induced localized states perturbed the BaSi_2_ bandgap and directly affected carrier transport and recombination. Specifically, silicon vacancies (V_Si_) introduced deep-level states that acted as strong Shockley–Read–Hall (SRH) recombination centers, significantly reducing minority-carrier lifetime (τ) and consequently lowering the open-circuit voltage (V_oc_) and fill factor (FF). Barium vacancies (V_Ba_) generated shallow acceptor states that primarily affected carrier concentration and mobility, exerting moderate influence on FF and minimal effect on V_oc_. BaSi antisite defects introduced shallow donor levels that slightly perturbed carrier transport but potentially increased SRH recombination under high defect densities, thereby reducing τ. According to previous reports, these qualitative relationships clarified how each defect type influenced device performance, while defect passivation strategies such as hydrogenation or dielectric coating effectively mitigated these effects [3,29,30]. Deep-level defects, particularly V_Si_, acted as strong SRH centers that limited minority-carrier lifetime, whereas shallow defects such as VBa and BaSi primarily affected mobility through Coulomb scattering.

**Figure 4:**
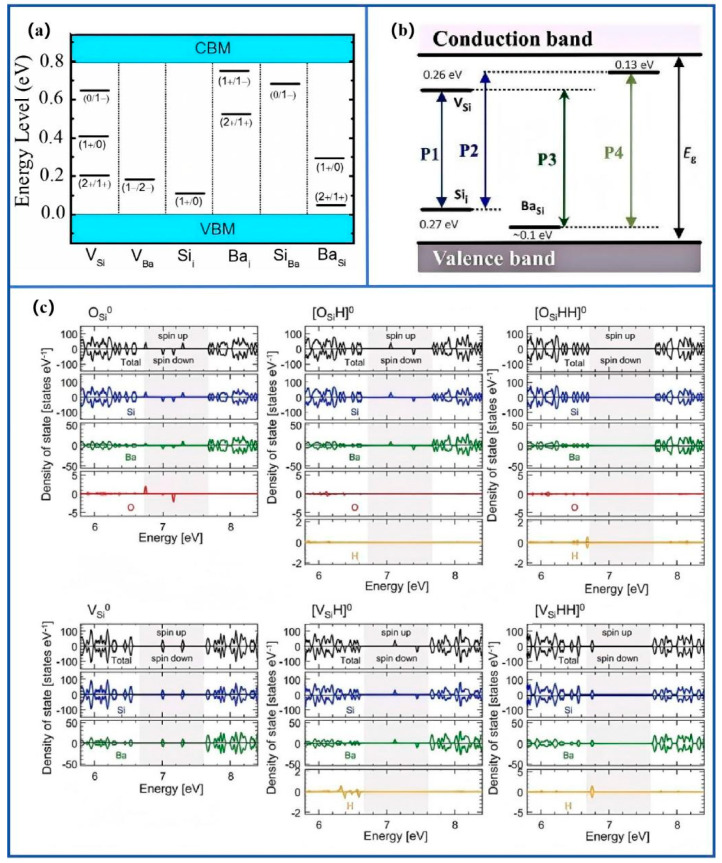
Electronic activity and radiative characteristics of intrinsic defects in BaSi_2_: (**a**) ionization levels within the DFT-calculated bandgap; (**b**) schematic model of radiative defect centers; (**c**) atom-resolved spin-polarized density of states illustrating defect-induced mid-gap states. Reproduced with permission. Refs. [29,35] Copyright 2022, Phys. Status Solidi A & Copyright 2024, ACTA MATER.

Sato, Mouesca, and Barra [29] combined EPR, photoluminescence, and DFT analyses and revealed that deep-level traps in BaSi_2_ predominantly originated from Si 3p orbital states associated with silicon vacancies (Figure 4c) [29,30]. Complementarily, Hara’s group [22,31,32,33,34] reported that post-deposition annealing modulated these electronic states by reducing oxygen-related traps and enhancing carrier lifetime. Their correlated optical and electrical studies provided direct evidence that hybridization between Ba 5d and Si 3p orbitals formed shallow acceptor states critical for p-type conductivity in doped BaSi_2_ epitaxial layers. The corresponding energy levels and radiative/non-radiative pathways were illustrated in Figure 4a,b [7,35]. Spin-polarized density-of-states (DOS) calculations (Figure 4c) [29,30] showed that deep levels originated from Si 3p orbitals, whereas shallow acceptor states arose from Ba 5d-Si 3p hybridization. Hall-effect measurements (Table 1) demonstrated that hydrogenation increased mobility from 25 to 1240 cm^2^·V^−1^·s^−1^ by neutralizing charged scattering centers, confirming the effectiveness of defect passivation.

Combined spectroscopic and theoretical analyses clarified the electronic origin of defect states in BaSi_2_. However, most studies emphasized intrinsic defects while neglecting dopant and grain-boundary effects. Discrepancies between DFT-predicted and experimentally observed levels highlighted the need for hybrid analytical approaches. Integration of advanced spectroscopic techniques with atomistic modeling could provide a more comprehensive understanding of carrier dynamics.

### 2.3. Defect–Optoelectronic Coupling

Photovoltaic performance depended on interactions between defect populations and optoelectronic processes. Open-circuit voltage (VOC), short-circuit current density (JSC), and fill factor (FF) quantified device performance, which depended on defect populations, carrier recombination, and transport properties. Deep-level defects provided dominant non-radiative recombination channels, thereby limiting VOC. Extended and interface defects created shunting paths and reduced the fill factor. Some shallow defects remained quasi-neutral, with effects dependent on growth conditions and doping [31,36].

Device stability was influenced by defect evolution under operational stress. Accelerated aging tests and in situ measurements (TEM, KPFM) revealed migration and clustering of defects, which altered local band structures and transport properties. Understanding these dynamics informed rational passivation strategies and deposition optimization aimed at approaching theoretical efficiency limits [32].

## 3. Passivation and Defect Control Strategies

### 3.1. Chemical and Structural Passivation Mechanisms

Defect management in BaSi_2_ thin films required an integrated nanoscale approach targeting point defects, extended defects, and interfacial states. In this context, “structural passivation” referred to the introduction of additional nanoscale layers or interphases composed of dielectric or chemically compatible thin films, which physically and chemically reduced defect density and stabilized the interface. Chemical passivation, primarily via hydrogenation and fluorination, operated through saturation of dangling bonds and electrostatic charge compensation [33,37]. Hydrogen passivation selectively targeted deep-level defects, including silicon vacancies (V_Si_) and BaSi antisites, forming defect-hydrogen complexes that efficiently neutralized recombination centers and enhanced carrier mobility. These hydrogenated complexes were, however, metastable, and partial de-passivation occurred above ~400 °C, limiting thermal stability. In contrast, fluorine passivation preferentially bound to shallow acceptor-like defects, such as barium vacancies (V_Ba_), leveraging its high electronegativity to stabilize charges at the interface and near-surface regions. Fluorinated bonds exhibited greater thermal stability than hydrogenated ones, allowing retention of passivation effects at elevated temperatures; however, excessive fluorine incorporation could induce Frenkel-type defects, narrowing the processing window [34,38,39,40].

Hall effect measurements (Table 1) demonstrated that hydrogenation increased mobility from 25 to 1240 cm^2^·V^−1^·s^−1^ by neutralizing charged scattering centers, confirming the effectiveness of defect passivation [35]. Benincasa, Hoshida, and Deng [3] demonstrated that atomic hydrogen effectively passivated deep defect levels in BaSi_2_ epitaxial films, significantly enhancing photoluminescence intensity and carrier lifetime. Hydrogen plasma treatment was demonstrated to neutralize silicon vacancies (V_Si_) and BaSi antisite defects by forming stable defect-hydrogen complexes. Nevertheless, these bonds exhibited metastability, leading to reversible de-passivation above 400 °C, which constrained thermal stability. Fluorination leveraged the high electronegativity of fluorine to enhance thermal retention through dipole-field-assisted charge compensation; however, supra-stoichiometric incorporation could induce Frenkel-type defects, thereby narrowing the permissible processing window [38,39].

Dielectric and interfacial passivation, such as via Atomic Layer Deposition (ALD) of Al_2_O_3_ or SiN_x_, provided both chemical and electrostatic passivation beyond mere physical isolation. Surface hydroxyl groups contributed to chemical passivation, while fixed charges induced field-effect passivation, collectively reducing interface state density [41,42]. Recent XPS studies showed that the formation of interface dipoles at ALD/SiN_x_-BaSi_2_ interfaces shifted the local vacuum level and modified band alignment, effectively reducing electron-hole recombination at the interface. The induced dipoles and resulting band bending contributed to a favorable energetic landscape for carrier extraction, highlighting the importance of interface engineering in addition to chemical passivation. Optimal dielectric thickness, generally 2–5 nm, balanced field modulation and carrier tunneling probability. In situ interphase reconstruction generated nanoscale layers, such as barium silicates or germanates, that simultaneously passivated interfacial and grain boundary defects [43]. Although these interphases improved environmental stability, they could introduce additional carrier transport barriers, necessitating precise thickness control below ~1–2 nm [44,45].

The heterogeneity of extended defects further complicated passivation design. Grain boundary engineering exploited chemical potential gradients to achieve preferential passivant segregation [46,47]. Advanced electron microscopy revealed that passivation efficacy varied with grain boundary type, exhibiting preferential segregation to high-energy random boundaries relative to low-Σ coincidence boundaries, determined by differences in segregation energies and local atomic coordination [48]. Despite these advances, establishing a standardized framework that correlated atomic-scale passivation mechanisms with device-level reliability remained essential. Hydrogenation and interface engineering proved effective in reducing deep-level defects, yet challenges remained in long-term stability and interfacial charge control. Most strategies remained empirical, with limited atomistic insight into hydrogen-defect interactions. Future work should focus on mechanistic understanding and thermally stable passivation schemes compatible with device processing.

### 3.2. Doping, Interface, and Epitaxial Regulation

The epitaxial growth conditions of BaSi_2_ films determined the intrinsic defect landscape. They controlled the effectiveness of subsequent passivation strategies. The crystalline quality, preferred orientation, and intrinsic defect density strongly depended on the Ba/Si flux ratio (R_Ba_/R_Si_), as revealed by RHEED and XRD analyses [49]. This ratio adjusted the chemical potential during epitaxial growth and altered the formation energies of intrinsic point defects. Near-stoichiometric growth reduced the density of recombination-active centers and formed a low-defect-density template suitable for interfacial passivation.

Additionally, the surface properties of different BaSi_2_ crystallographic planes, including surface energy and surface electronic states, played a critical role in defect formation and passivation behavior. Table 2 summarized the surface energy and the positions of hole and electron states for various BaSi_2_ surfaces, providing a concise overview of surface-dependent electronic characteristics relevant to growth and interface engineering [11].

**Figure 5:**
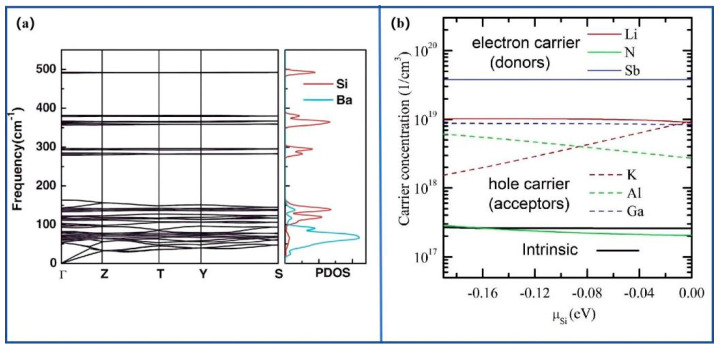
Phonon dispersion and carrier modulation in BaSi_2_: (**a**) phonon band structure and partial density of states; (**b**) equilibrium carrier concentrations of doped BaSi_2_ as a function of Si chemical potential, with intrinsic behavior shown for comparison. Reproduced with permission. Ref. [7] Copyright 2020, JJAP.

Figure 5 showed the effect of dopant incorporation on the physical properties of BaSi_2_ [7,50]. Figure 5a presented the phonon band structure and partial density of states. The results confirmed the lattice dynamical stability of BaSi_2_ and indicated that doping slightly perturbed the phonon modes. This change affected carrier scattering and defect-related recombination. Figure 5b showed the equilibrium carrier concentrations of doped BaSi_2_ as a function of the Si chemical potential. The data demonstrated that controlled doping modulated the electronic environment and carrier density. Optimized doping adjusted carrier transport and modified intrinsic defect energetics. When combined with epitaxial and chemical passivation, it improved carrier lifetime and photovoltaic performance.

Hybrid passivation strategies that combined ALD-derived dielectric layers with hydrogen plasma treatment showed complementary effects. They reduced both surface states and bulk or grain-boundary defects. The improvement in optoelectronic properties depended on epitaxial quality, process sequence, and interfacial reaction kinetics [51,52].

### 3.3. Evaluation of Passivation Effectiveness

A systematic evaluation framework was essential for assessing passivation efficacy in BaSi2 thin films [53]. This framework integrated electronic metrics, including interface state density, quasi-Fermi level splitting, and carrier lifetime; device performance parameters, such as ΔVOC, fill factor, and long-wavelength EQE; and stability under accelerated aging conditions (85 °C/85% RH, light soaking) [54,55].

Thermal and operational stability remained a critical challenge. Hydrogen de-passivation, trap migration within ALD dielectrics, and interfacial phase transformations degraded device performance. In particular, defect migration and clustering locally increased non-radiative recombination centers, reduced carrier lifetime (τ), and generated leakage paths that negatively impacted Voc and FF. Oxidation at grain boundaries or interfaces introduced additional trap states or altered local electronic structure, further compromising device reliability. Elucidating these mechanisms required in situ characterization techniques, including environmental transmission electron microscopy (TEM), X-ray absorption fine structure (XAFS), X-ray photoelectron spectroscopy (XPS), and bias-dependent photoluminescence (PL) or deep-level transient spectroscopy (DLTS), to monitor passivant evolution under operational stress [56]. The multiscale heterogeneity of interfaces and grain boundaries further necessitated high-spatial-resolution spectroscopy combined with nanoscale electrical mapping, such as electron-beam-induced current (EBIC) analysis, to evaluate passivation penetration and durability.

Industrial applicability further constrained technique selection. Plasma or ion-beam treatments were effective at laboratory scale but could induce thin-film damage. ALD and low-temperature CVD provided reproducible passivation but involved high precursor costs and stress management challenges [57,58]. Molecular-layer or two-dimensional coverage strategies enhanced surface uniformity but required precise integration with downstream electrodes. For clarity, Table 3 summarized the cost, throughput, and process compatibility of these common passivation methods, providing guidance for industrial implementation.

Future advances were anticipated from atomic-scale integration of passivation within epitaxial growth, multiscale gradient or layered passivation designs targeting distinct defect types, and data-driven optimization leveraging machine learning and multiscale simulations. Such interdisciplinary approaches facilitated the transition from empirical optimization to predictive design, guiding the development of robust, scalable passivation strategies for BaSi_2_ thin-film photovoltaics.

## 4. Conclusions

The performance of BaSi2 thin-film photovoltaics was intrinsically governed by the interplay between crystallographic defects and their electronic activity. Point defects, extended defects, and interfacial states collectively modulated carrier transport and recombination kinetics, thereby imposing fundamental limits on photovoltaic conversion efficiency, open-circuit voltage, and fill factor. Nanoscale passivation strategies, including chemical, dielectric, and interfacial engineering, mitigated defect-induced performance degradation, with efficacy strongly determined by the initial epitaxial quality and microstructural features. Atomic- and nanoscale control of defects, grain boundaries, and interfacial layers was critical for optimizing carrier lifetime, mobility, and overall device performance. Nevertheless, comprehensive understanding of the synergistic interactions among distinct defect types, their dynamic evolution under operational stress, and the long-term stability of passivation remained incomplete. Addressing these challenges required the integration of in situ characterization, multiscale modeling, and data-driven approaches to establish predictive strategies for defect regulation, interface engineering, and microstructural optimization, thereby providing a rational basis for advancing the efficiency, stability, and scalability of BaSi_2_ thin-film photovoltaics and other emerging thin-film solar materials. Future progress was anticipated through developing novel passivation materials and multilayer interfaces to enhance carrier lifetime and thermal stability, implementing machine learning-guided growth and post-treatment protocols to predict and minimize defect densities, exploring alloying or doping strategies to fine-tune electronic properties and suppress deep-level recombination, and scaling up epitaxial or hybrid thin-film fabrication techniques compatible with industrial processes to ensure reproducibility and cost-effectiveness. These approaches could overcome current limitations and accelerate the practical deployment of BaSi_2_-based photovoltaic devices.

## Figures and Tables

**Table 1:** Electrical transport characteristics of BaSi2 samples with different hydrogenation levels (tBaSi:H): conductivity type, carrier concentration, and Hall mobility. Reproduced with permission. Ref. [35] Copyright 2022, Phys. Status Solidi A.

tBaSi:H	Type of Conductivity	Concentration (cm^−3^)	Mobility (cm^2^ V^−1^ s^−1^)
1 min	p	1.1 × 10^17^	25
15 min	p	1.8 × 10^17^	97
30 min	n	9 × 10^15^	1240

**Table 2:** Surface energy and surface electronic states of different BaSi_2_ crystallographic planes. Reproduced with permission. Ref. [11] Copyright 2017, Jpn. J. Appl. Phys.

Surface	Surface Energy (meV/Å^2^)	Hole Surface State (eV Above VBM)	Electron Surface State (eV Below CBM)
(001)	40.0	One deep	One deep
(010)	29.0	One shallow	One shallow
(100)	29.3	No	One shallow at 0.05
(011)	40.0	One shallow	One shallow
(101)	34.6	Surface band at 0.43	No
(110)	40.1	Two deep	One shallow
(111)	28.8	No	No

**Table 3:** Comparison of passivation methods for industrial scalability.

Method	Cost	Throughput	Process Compatibility/Challenges
ALD	High	Moderate	Excellent uniformity, precise thickness control, precursor cost and stress management required
Plasma/Ion-beam	Low	Low	Rapid treatment, may induce thin-film damage, limited wafer-scale reproducibility
Molecular-layer/2D coverage	Moderate	Moderate	Enhances surface uniformity, requires precise integration with electrodes, moderate equipment cost

## Data Availability

Not applicable.
